# Schizophrenia polygenic risk predicts general cognitive deficit but not cognitive decline in healthy older adults

**DOI:** 10.1038/s41398-020-01114-8

**Published:** 2020-12-08

**Authors:** Adrianna P. Kępińska, James H. MacCabe, Dorina Cadar, Andrew Steptoe, Robin M. Murray, Olesya Ajnakina

**Affiliations:** 1 Department of Psychosis Studies, Institute of Psychiatry, Psychology and Neuroscience, King’s College London, London, UK; 2 Department of Behavioural Science and Health, Institute of Epidemiology and Health Care, University College London, London, UK; 3 Department of Biostatistics & Health Informatics, Institute of Psychiatry, Psychology and Neuroscience, King’s College London, University of London, London, UK

**Keywords:** Diagnostic markers, Clinical genetics

## Abstract

There has been a long argument over whether schizophrenia is a neurodegenerative disorder associated with progressive cognitive impairment. Given high heritability of schizophrenia, ascertaning if genetic susceptibility to schizophrenia is also associated with cognitive decline in healthy people would support the view that schizophrenia leads to an accelerated cognitive decline. Using the population representative sample of 6817 adults aged >50 years from the English Longitudinal Study of Ageing, we investigated associations between the biennial rate of decline in cognitive ability and the schizophrenia polygenic score (SZ-PGS) during the 10-year follow-up period. SZ-PGS was calculated based on summary statistics from the Schizophrenia Working Group of the Psychiatric Genomics Consortium. Cognition was measured sequentially across four time points using verbal memory and semantic fluency tests. The average baseline verbal memory was 10.4 (SD = 3.4) and semantic fluency was 20.7 (SD = 6.3). One standard deviation (1-SD) increase in SZ-PGS was associated with lower baseline semantic fluency (β = −0.25, 95%CI = −0.40 to −0.10, *p* = 0.002); this association was significant in men (β = −0.36, 95%CI = −0.59 to −0.12, *p* = 0.003) and in those who were aged 60–69 years old (β = −0.32, 95%CI = −0.58 to −0.05, *p* = 0.019). Similarly, 1-SD increase in SZ-PGS was associated with lower verbal memory score at baseline in men only (β = −0.12, 95%CI = −0.23 to −0.01, *p* = 0.040). However, SZ-PGS was not associated with a greater rate of decline in these cognitive domains during the 10-year follow-up. Our findings highlight that while genetic susceptibility to schizophrenia conveys developmental cognitive deficit, it is not associated with an ongoing cognitive decline, at least in later life. These results do not support the neo-Kraepelinian notion of schizophrenia as a genetically determined progressively deteriorating brain disease.

## Introduction

The question of whether schizophrenia is a neurodegenerative disorder associated with progressive cognitive impairment has been debated for over a century. Many psychiatrists, with support from neuropsychological^1,2^ and neuroimaging^3,4^ reports, believe that the course of schizophrenia is characterised by cognitive decline^5,6^. However, prospective longitudinal studies are scarce. A small decline in cognitive function over a 10-year period following the onset of schizophrenia was reported^7^, but the authors could not be certain whether this was due to some intrinsic schizophrenia process(es) or resulted from adverse effects associated with its treatment. There are some suggestions that schizophrenia-related cognitive decline may be particularly pronounced in later life^8,9^. Indeed, an 18-year follow-up study showed that cognition in individuals with a psychotic disorder declined on all but 2 tests compared with controls, with the largest effect among participants who were 50 years or older^10^.

Schizophrenia is highly heritable^11^, with a polygenic architecture^12^. Recent evidence suggests that the polygenetic underpinning of schizophrenia overlaps with general cognitive ability^13,14^. Family studies also highlight the presence of cognitive impairments in schizophrenia patients prior to the onset of schizophrenia symptoms^15^, and in their unaffected relatives but in more attenuated forms^16^. Therefore, one of the possible ways to test the view that schizophrenia leads to a greater rate of decline in cognition may be through ascertaining whether genetic susceptibility to schizophrenia is also associated with cognitive decline in healthy people using the polygenic score (PGS) approach. PGS for schizophrenia encompasses multiple common genetic variants of the small effect associated with the illness that are scattered across the whole genome^17^, and as such, it indexes susceptibility for this disease^18^. Although several longitudinal studies have attempted to establish the effect of the PGS on cognition^19–21^, they produced mixed results, possibly due to small samples, restricted accountability of important confounders, or limited follow-up time-points with intervals ranging over several decades when measuring cognitive changes in the general population. These inconsistent findings reinforce uncertainty concerning the origins of cognitive impairment in those with schizophrenia.

Therefore, using a large, phenotypically well-defined sample of population-representative older adults, we investigated whether common genetic variants associated with schizophrenia additively confer a stable deficit in cognitive ability, measured sequentially across four time points, or a greater risk of accelerated cognitive decline, or both, over the 10-year follow-up. Assuming a variation in cognitive impairment in schizophrenia is a function of the degree of genetic liability to the disorder, we hypothesised that polygenic score for schizophrenia would be significantly associated with lower cognition at baseline and a greater rate of decline in cognition during follow-up in healthy adults. Additionally, given age and gender differences in cognitive functions and schizophrenia risk^22–24^, we also investigated whether the potential relationships of a polygenic score for schizophrenia with cognition at baseline and during follow-up in healthy adults differed by age and gender.

## Methods

### Sample

We utilised data from the English Longitudinal Study of Ageing (ELSA), which is an ongoing large, multidisciplinary study of a nationally representative sample of the English population aged ≥50 years. The ELSA study started in 2002 (wave 1), with participants recruited from an annual cross-sectional survey that is designed to monitor the health of the general population. The ELSA sample was then followed-up every 2 years. For the present study, baseline data were obtained from either wave 2 (2004–2005) for the core members who started at wave 1, or wave 4 for the participants joining the study at wave 4 through the refreshment sample; the included participants took part in the blood draws during home visits by a nurse. Follow-up data were ascertained from waves 4 (2009–2010) to wave 8 (2016–2017), which is the latest wave of data collection. We excluded participants with diagnosed organic causes of cognitive decline, such as history of dementia and stroke at baseline as well as those with a previous diagnosis of schizophrenia. Those ELSA participants who were included in the study or excluded from the final cohort did not differ in terms of age at baseline; however, the former group included participants with a higher educational attainment, higher accumulated wealth, lower proportion of people with a long-standing limiting health condition, depressive symptoms and smokers compared those respondents who were excluded for relevant variables in this study (Supplementary Table 1). Ethical approval for each of the ELSA waves was granted by the National Research Ethics Service (London Multicentre Research Ethics Committee). All participants gave informed consent.

### Study variables

#### Cognition

**Fig. 1:**
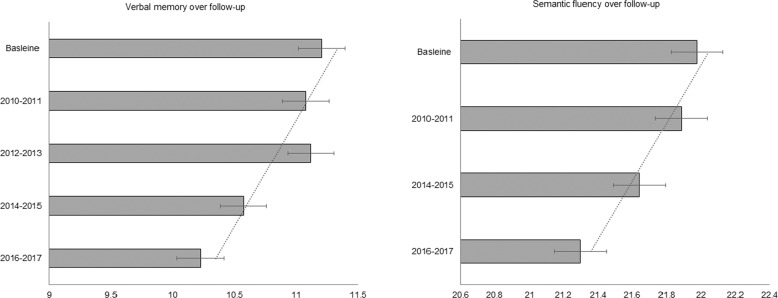
Distribution of the cognitive domains across all waves of data collection for the 6817 ELSA participants included in this study presented as means and standard deviations. The baseline data were obtained from either wave 2 (2004–2005) for the core members who started at wave 1, or wave 4 for the participants joining the study at wave 4 through the refreshment sample. Follow-up data were ascertained from waves 4 (2009–2010) to wave 8 (2016–2017), which is the latest wave of data collection.

Cognition was measured employing tests for verbal memory and semantic fluency, which were chosen because both these cognitive domains are important predictors of clinically significant cognitive decline in healthy older adults^25^. To measure verbal memory, immediate and delayed verbal memory were assessed using a word-learning task, which entailed recalling as many out of 10 common words that were read out to them as possible immediately and after a short delay during which they completed other cognitive tests^26^. Following the protocol of previous studies^27^, the results for immediate and delayed recall were then combined to give an overall verbal memory variable measured on a continuum from 0 to 10 with a higher score indicating a better memory performance. Semantic fluency was measured with a verbal fluency test, where participants were asked to think of as many animal names as they could in 1 min. The total number of animal names written by participants was used as a continuous measure of semantic fluency; the semantic fluency score was measured on a continuum from 0 to 20 with a higher score indicating better performance. Although this task primarily focused on semantic fluency, it combined various aspects of broader executive function including cognitive flexibility, processing speed, inhibitory control, and verbal fluency. As semantic fluency test was not administered at wave 6, in line with previous work in this area^26^, there was a longer follow-up gap in the assessment of this cognitive domain from wave 5 (2010–2011) to wave 7 (2014–2015) (Fig. 1). The distribution of these cognitive domains across all waves of data collection is provided in Supplementary Table 2.

#### Covariates

We included an extensive set of covariates encompassing different aspects of persons’ health and life, which have been shown to affect cognitive function of older English people^28^. Demographic covariates include gender (male) and age; to capture non-linear aging effects which cognition is susceptible to, we also included age^2^ as a covariate. Social determinants, such as wealth (poorest, middle and wealthiest as reference) and educational attainment^29^, were also included as covariates. To reflect the accumulation of resources at older ages, wealth was measured at baseline by summing wealth from property, possessions, housing, investments, savings, artwork, jewellery and net of debt^30^. The wealth variable was further divided into tertile to represent the groups of individuals with the high, intermediate and low levels of wealth. Educational attainment was measured with how many years of schooling each participant completed^31^. As comorbid health conditions can affect cognitive ageing, we included depressive symptoms and the presence of a limiting long-standing illness as covariates. The presence of a limiting long-standing illness was measured with the participants’ reporting presence of any limiting health illness (coded as “yes”), or absence of any limiting illnesses or health conditions (coded as “no”). Depressive symptoms were measured with an 8-item version of the Centre for Epidemiologic Studies Depression Scale^32^, which has been found to have comparable psychometric properties to the full 20-item scale;^33^ a score ≥4 was used to define participants with severe depressive symptoms^34^. Behavioural risk factors included smoking status (current smoker and non-smoker was a reference). Because the ε4 allele of the apolipoprotein E gene (APOE-ε4) has previously been associated with cognitive decline in normal aging^35^, we adjusted our analyses for APOE-ε4. Consistent with previous research^36^, APOE-ε4 status was defined according to the absence (APOE ε2/2, ε2/3 and ε3/3) or presence (APOE ε2/4, ε3/4 and ε4/4) of APOE-ε4 alleles. Lastly, genetic ancestry (as was measured with principal components (see below)), was included as covariates to account for any ancestry differences in genetic structures that could bias our results^37^.

### Genetic data

#### Quality control

The genome-wide genotyping was performed at University College London Genomics in 2013-2014 using the Illumina HumanOmni2.5 BeadChips (HumanOmni2.5-4v1, HumanOmni2.5-8v1.3). Single-nucleotide polymorphism (SNPs) were excluded if they were non-autosomal, the minor allele frequency was <0.01%, if more than 2% of genotype data were missing and if the Hardy-Weinberg Equilibrium *p*-value<10^−4^. Samples were removed based on call rate (<0.99), suspected non-European ancestry, sex difference in allelic frequency of ≥0.2, heterozygosity and relatedness. We employed the principal components analysis^37^ to identify those individuals who deviated from European ancestry (i.e., ethnic outliers). This set of analyses demonstrated the presence of ancestral admixture in the 65 individuals, who were subsequently removed; individuals who self-reported they were of non-white ethnicity were also removed. Using the updated sample, we calculated principal component (PCs) (Supplementary Fig. 1), which then were used to adjust for possible population stratification in the association analyses^37,38^.

#### Polygenic score (PGS) analyses

PGS for schizophrenia (SZ-PGS) was calculated using summary statistics from genome-wide association study (GWAS) conducted by the Schizophrenia Working Group of the Psychiatric Genomics Consortium (PGC)^39^. Schizophrenia-associated SNPs, weighted by their effect size derived from the PGC, were summed in a continuous score using PRSice^40^ following specifications outlined previously^41^. As previous research highlighted that PGSs built from directly genotyped data either had more predictive power^31^ or did not differ significantly from PGSs calculated using imputed data^41^, we calculated PGSs based on genotyped data at different *p*-value cut-offs. Because PGSs including all available SNPs either explain the most amount of variation in a trait or are not significantly different than PGSs based on different *p*-value thresholds^41^, we utilised PGS that was based on a threshold of *p*-value of 1. To aid the interpretability of the results, SZ-PGS was standardised to a mean of 0 (SD = 1).

### Statistical analysis

#### Association analyses

To assess the relationships of SZ-PGS with the general cognition and the rate of change in cognitive ability during the 10-year follow-up, we employed linear mixed effect models (LMMs) with maximum likelihood estimation^42^. LMMs have been shown to be useful for the analysis of longitudinal data with an evitable loss to follow-up data^43^. Having considered linear, quadratic and cubic LMMs, Akaike Information Criterion and Bayesian Information Criterion^44,45^ showed that the linear model was the most appropriate for our analyses. To investigate whether age influenced the potential relationships between SZ-PGS and cognitive decline over follow-up, we stratified our analyses by age groups, which were formed based on tertiles results (i.e., 50–59 years, 60–69 years and ≥70 years). Given the previously observed gender differences in cognitive functions^22^, we additionally performed sex-stratified analyses. We used a significance level of 0.05 (two-tailed) for all analyses. All analyses were conducted in STATA release 14 (STATA Corp LP, USA).

#### Calculate power and predictive accuracy of a polygenic score

To investigate whether SZ-PGS included in the present study had sufficient power to detect relationships with cognition at baseline and a greater rate of decline in cognition during follow-up in healthy adults, we estimated the power of the calculated SZ-PGS using the Avengeme package implemented in R^18^. This showed that SZ-PGS had sufficient power for the subsequent analyses (power=1, *p* = 6.26 × 10^−17^).

## Results

### Sample characteristics

The baseline demographic and health characteristics of the total sample are presented in Table 1. The sample comprised 6817 individuals with a baseline mean age of 64.3 years old (standard deviation (SD) = 9.3, range = 50–101); 25.3% (*n* = 1724) of participants were APOE-ε4 carriers, 46.2% (*n* = 3159) were men, 30.9% (*n* = 2108) were unmarried and 32.5% (*n* = 2154) had a low level of accumulated wealth. The average baseline memory score was 10.4 (SD = 3.4) and the executive function score was 20.7 (SD = 6.3).

**Table 1:** Sample characteristics at baseline.

Baseline sample characteristics	Total sample *n* = 6817
	*N* (%)/Mean (SD)
Age at baseline (years)	64.6 (9.3)
Age groups	
50–59 years	2487 (36.5)
60–69 years	2332 (34.2)
≥70 years	1998 (29.3)
Gender	
Female	3667 (53.8)
Male	3159 (46.2)
APOE-ε4 present	1724 (25.3)
Currently smoker	978 (15.2)
Married	2108 (30.9)
Wealth	
Low	2154 (32.5)
Intermediate	2234 (33.6)
High	2251 (33.9)
Educational attainment (years)	13.7 (3.8)
Limited life health condition (any)	2132 (31.3)
Depression diagnosis	735 (13.3)
Cognition	
Memory score	10.4 (3.4)
Executive function score	20.7 (6.3)

*APOE-ε4* two *ε4* alleles of the Apolipoprotein E gene, *SD* standard deviation.

### Rate of change in cognition over the 10-year follow-up

The rate of decline in cognition over the 10-year follow-up is depicted in Fig. 1. The average biennial rate of decline in verbal memory during the 10 years was 1.21 points (95%CI = 0.99–1.44, *p* < 0.001). For semantic fluency, the average biennial rate of decline was 1.36 points (95%CI = 1.07–1.65, *p* < 0.001) over the follow-up period.

### SZ-PGS and verbal memory in older adults

There was a trend towards a statistically significant relationship of one standard deviation (1-SD) increase in SZ-PGS with a lower baseline verbal memory score (β = −0.07, 95%CI = −0.14–0.01, *p* = 0.091) (Table 2). When the analyses were stratified by gender, the results showed that 1-SD increase in SZ-PGS was associated with lower verbal memory score in men (β = −0.12, 95%CI = −0.23 to −0.01, *p* = 0.040) but not in women (Supplementary Table 3) highlighting a significant interaction with gender. There was also a trend towards significance in the relationship of SZ-PGS with verbal memory score in older adults who were aged 70 years old and older (β = −0.14, 95%CI = −0.28–0.004, *p* = 0.056) (Supplementary Table 4). Nonetheless, there was no significant association between SZ-PGS and the rate of decline in verbal memory during the 10-year follow-up period.

**Table 2:** Associations between schizophrenia polygenic score (SZ-PGS) and cognitive function in older adults over the 10-year follow-up.

	Verbal memory		Semantic fluency	
	β (95%CI)	*P*-value	β (95%CI)	*P-*value
*Baseline*				
SZ-PGS	−0.07 (−0.14–0.01)	0.091	−0.25 (−0.40 to −0.09)	0.002
Age	−0.14 (−0.15 to −0.14)	<0.001	−0.19 (−0.21 to −0.18)	<0.001
Gender	−1.08 (−1.23 to −0.92)	<0.001	0.02 (−0.29 to 0.33)	0.906
Current smoker	−0.10 (−0.33–0.13)	0.386	−0.58 (−1.04 to −0.12)	0.013
Low level of wealth	−0.60 (−0.77 to −0.42)	<0.001	−0.62 (−0.97 to −0.27)	<0.001
Education attainment	0.19 (0.17–0.21)	<0.001	0.43 (0.38 to 0.47)	<0.001
Depression diagnosis	−0.53 (−0.77 to −0.30)	<0.001	−0.81 (−1.28 to −0.33)	0.001
*APOE-ε4* present	−0.27 (−0.45 to −0.10)	0.002	−0.31 (−0.66 to 0.04)	0.074
Limiting health conditions (any)	−0.18 (−0.35 to −0.01)	0.041	−0.17 (−0.66 to 0.04)	0.320
*Rate of change*				
SZ-PGS	0.003 (−0.01–0.02)	0.741	0.01 (−0.02–0.03)	0.740
Age	−0.06 (−0.03–−0.09)	<0.001	0.06 (0.002–0.11)	0.044
Gender	−0.02 (−0.05–0.01)	00.285	−0.06 (−0.12 to −0.002)	0.041
Current smoker	−0.07 (−0.12–−0.02)	0.002	−0.05 (−0.14–0.03)	0.229
Low level of wealth	−0.01 (−0.04–0.03)	0.707	−0.08 (−0.14 to −0.01)	0.028
Education attainment	0.002 (−0.002–0.01)	0.255	−0.01 (−0.01–0.002)	0.121
Depression diagnosis	−0.01 (−0.06–0.04)	0.629	−0.05 (−0.15 to 0.04)	0.258
*APOE-ε4* present	−0.08 (−0.12–−0.04)	<0.001	−0.10 (−0.16 to −0.03)	0.005
Limiting health conditions (any)	−0.03 (−0.06–0.01)	0.126	−0.11 (−0.18 to −0.05)	0.001
*Variance*^a^				
Within-person	0.07 (0.06–0.08)		0.16 (0.13–0.21)	
In initial status	3.69 (3.66–4.28)		15.99 (14.77–17.31)	
In rate of change	0.03 (−0.02–0.08)		0.08 (−0.09–0.26)	

The models were further adjusted for age2 to capture non-linear aging effects of which cognition is susceptible to and 4 principal components to account for any ancestry differences in genetic structures that could bias the results.

*CI* confidence intervals, *SZ-PGS* polygenic score for schizophrenia, *APOE-ε4* two *ε4* alleles of the Apolipoprotein E gene.

^a^The within-person variance is the overall residual variance in cognition that is not explained by the model. The initial status variance component is the variance of individuals’ intercepts about the intercept of the average person. The rate of change variance component is the variance of individual slopes about the slope of the average person.

### SZ-PGS and semantic fluency in older adults

1-SD increase in SZ-PGS was associated with lower sematic fluency at baseline (β = −0.25, 95%CI = −0.40–0.09, *p* = 0.002). Further analyses showed this association was significant in men (β = −0.36, 95%CI = −0.59 to −0.12, *p* = 0.003) (Supplementary Table 3) and in those who were aged 60–69 years old (β = −0.32, 95%CI = −0.58 to −0.05, *p* = 0.019) (Supplementary Table 5). Although APOE-ε4, tobacco smoking, educational attainment and lower wealth were inversely associated with semantic fluency during follow-up, SZ-PGS was not associated with a greater rate of decline in these cognitive domains during the 10-year follow-up period.

## Discussion

In the present study, we investigated the relationships of multiple common genetic variants for schizophrenia, which additively indexed susceptibility for this illness^18^, with cognition and rate of decline in cognition during the 10-year follow-up period in a large population-representative sample of older adults. At the core of the study was the notion that if cognitive impairment in schizophrenia is a consequence of genetic liability to the disorder, with greater impairment indicating greater liability, the association between the genetic liability to schizophrenia and cognitive decline would be observed in non-psychotic adults.

In support of the previous findings highlighting molecular genetic overlap between general cognitive ability and risk for schizophrenia in the general population^13,14^, we found that SZ-PGS was significantly associated with lower verbal memory and semantic fluency scores at baseline in men independently from demographic factors, health-related factors and APOE-ε4 status. This is consistent with earlier twin studies which highlighted that boys had higher heritability for a verbal measure of cognitive ability compared to girls^46,47^, in turn highlighting that this genetic propensity to a higher verbal measure of cognitive ability in men extends to later lifeAdditive contribution of common genetic markers for schizophrenia was also significantly higher for semantic fluency score among adults who were aged 60–69-year old, supporting the notion that genetic influence on sematic fluency differs by age groups^48^.

In contrast to our hypothesis, however, common genetic variants associated with schizophrenia additively did not confer a greater rate of decline in cognition during the 10-year follow-up in older people from the general population. Although some evidence for gender differences in cognitive aging exist, as further supported by our results, these were not associated with genetic propensity for schizophrenia. The fact that we observed a significant cognitive decline during the follow-up period linked to the effects of APOE-ε4, tobacco smoking, educational attainment and lower wealth, demonstrates our study had the capacity to capture the cognitive decline over time in community-dwelling older adults. Consequently, our results contradict McIntosh et al.^20^ who, having measured cognitive changes between ages 11 and approximately 70 in a large birth cohort, suggested that common genetic variants for schizophrenia accelerate cognitive ageing among non-psychotic individuals. As the timing and trajectory of these changes were unknown, it is possible such deficits may have arisen during adolescent rather than in later life^49^. Indeed, cognitive deficits have been shown to be present at a young age in some children who later developed schizophrenia^50^. The young children who are destined to develop schizophrenia show slower cognitive development in a range of cognitive domains, which results in further divergence in cognitive ability by the time schizophrenia develops^50^. However, evidence for the subsequent long-term cognitive decline during the transition to schizophrenia or following its onset is lacking^51,52^. In a subsequent analysis conducted by Ritchie et al.^21^ on the same sample as McIntosh et al.^20^ but capturing individuals aged 70–79-year old, a significant inverse relationship of schizophrenia polygenic score with general cognitive slope was observed. Although this finding appears to support a genetic overlap between cognitive decline and schizophrenia, this relationship did not survive multiple-comparisons correction. Since Ritchie et al.^21^ measured general cognition using the block-design task in contrast to the present verbal memory tasks, this may be a source of discrepancy between the studies. Nonetheless, our findings are further consistent with the results reported by Liebers et al.^19^ who found an association between SZ-PGS and cognitive deficit at age 60 years in 8616 community-dwelling individuals, but did not find a relationship between SZ-PGS and cognitive decline over the next decade.

There is growing interest in improving cognitive functions in patients with schizophrenia through pharmacological treatment or cognitive rehabilitation programmes. Our findings raise the possibility that the cognitive decline reported in schizophrenia patients may be due to other factors rather than intrinsic genetically determined schizophrenia process(es). The causes of cognitive decline in schizophrenia may include factors secondary to illness-related behaviours, such as substance abuse, cigarette smoking, poor nutrition as well as exposure to medications used in treating the disorder, such as antipsychotics, anticholinergics, and benzodiazepines^49^. Antipsychotic medications may also explain the brain changes, such as cortical gray matter reductions^53^, which in turn have been used as evidence that this illness is a progressive brain disorder^52^. Other factors including decreased environmental stimuli related to social isolation might also play a role in reducing cognitive capacity in patients with schizophrenia^52^. Certainly, there is evidence suggesting that loneliness contributes to a worsening in memory and verbal fluency over a decade in the general population^26^.

Cumulatively, our results do not support the neo-Kraepelinian notion of schizophrenia as a genetically determined progressively deteriorating brain disease, at least at the molecular level. Therefore, it may be important for optimum clinical care to reconsider the idea of the existence of intrinsically malignant process(es) underlying schizophrenia. This has contributed to an undue pessimism among mental health professionals and their consequent alienation from sufferers and their representatives, who increasingly advocate for the “recovery model”^49^. Of course, this is not to negate the serious and disabling problems that many patients with schizophrenia experience. Nonetheless, it is still possible that only a subset of the genetic factors for schizophrenia drive cognitive decline observed in patients with this illness, which, due to the nature of the PGS approach, might not have been captured in the present study. Therefore, further analyses, such as pathway-specific polygenic score analyses, genomic structural equation modelling and gene-set enrichment analyses, may be needed before we can draw more precise conclusions of the role schizophrenia risk loci may play in general cognition.

### Methodological considerations

We analysed a large nationally-representative cohort of older adults in England who were followed-up every two years. We further benefitted from the availability of repeated measures of cognition across a 10-year span. Our study included a relatively equal proportion of women and men from socio-economically diverse backgrounds. Confidence in these findings is strengthened by the use of a linear mixed model, which is an optimal way to identify the change in continuous dependent variables over time and quantify its association with a range of independent variables, all the while taking the intra-individual and inter-individual variation into account. To avoid providing results of questionable theoretical relevance^51^, we did not present our results as composite scores for broad cognitive functions; instead, we explored verbal memory and semantic fluency separately. The comprehensive inclusion of covariates in the analyses meant that we could control for identified confounding variables reducing any potential risk for biases in our results.

Nonetheless, several methodological limitations warrant a discussion. Although PGSs have the potential to improve health outcomes through their eventual implementation as clinical biomarkers, the poor generalisability of genetic studies across populations is noteworthy^54^. This is because the construction of PGSs is largely dependent on the availability of the summary statistics from genome-wide association studies (GWASs), which are currently predominately based on European participants^54^. Given genetic risk is different in European and non-European individuals^54^, further work is necessary to develop PGSs models in non-white populations. Similarly, by design, polygenic scores do not capture other structural variants beyond common genetic markers of relatively small effects, such as rare variants, poorly tagged or multiple independent variants, gen-by-gene interactions and gene-environment correlation^55^. Therefore, these factors will not be accounted for when applying PGSs in the analyses. Further, the lack of a pre-registered analytical protocol is a notable limitation of the present study. The presence of practice effects may have limited the observed decline in cognitive abilities. However, in line with the previous evidence^56^, the potential practice effects may be insignificant given the relatively long interval between the assessments of cognitive domains (i.e., 2 years). Finally, we assessed several associations, which may raise some concerns over multiple statistical testing. Considering our sample size was large enough to withstand multiple testing without increasing risk for false positive results and the fact that adjusting for multiple statistical testing has significant disadvantages^57^, rather than adjusting our *p*-values for multiple-testing, we followed the new guidelines for statistical reporting^58^ when presenting the results in the present study.

## Conclusion

Our findings highlight that while genetic susceptibility to schizophrenia conveys developmental cognitive deficit, it is not associated with an ongoing cognitive decline, at least in later life. Thus, our results do not provide support for the neo-Kraepelinian notion of schizophrenia as a genetically determined progressively deteriorating brain disease.

## Supplementary information

Supplementary material

## Data Availability

The ELSA data are available in public, open-access repository (the UK Data Archive) which is freely available and can be accessed at https://discover.ukdataservice.ac.uk.
