# Supplementary material for: Schizophrenia polygenic risk predicts general cognitive deficit but not cognitive decline in healthy older adults
Source: Transl Psychiatry. 2020 Dec 8;10:422. doi: 10.1038/s41398-020-01114-8 (PMC7722936; doi:10.1038/s41398-020-01114-8)

**Supplementary Table 1. Comparisons between the ELSA participants who were included in the analyses and those who were excluded**

|  |  |  | **Excluded (N=6125)** | **Included (N=6817)** |  |
| --- | --- | --- | --- | --- | --- |
|  |  |  | Mean (SD) / N(%) | Mean (SD) / N(%) | Statistics |
| *Socio-demographic characteristics* | |  |  |  |  |
|  | Age (years) |  | 64.53 (10.94) | 64.63 (9.46) | *t*=-0.53, df=12617, *p=*0.5970 |
|  | Gender (male) |  | 2615 (42.69) | 3094 (45.93) | x^2^=13.57, df=1, *p*<0.001 |
|  | Education (years) |  | 13.44 (3.80) | 13.72 (3.79) | *t*=-3.97, df=11587, *p<*0.001 |
|  | Accumulated wealth |  |  |  | x^2^=91.43, df=2, *p*<0.001 |
|  | High |  | 1713 (29.94) | 2332 (35.54) |  |
|  | Intermediate |  | 1847 (32.28) | 2272 (34.62) |  |
|  | Low |  | 2161 (37.77) | 1958 (29.84) |  |
|  |  |  |  |  |  |
| *Comorbid health issues* | |  |  |  |  |
|  | Limiting health conditions |  | 2307 (38.27) | 2114 (31.38) | x^2^=66.66, df=1, *p*<0.001 |
|  | Depression (score ≥4) |  | 698 (18.89) | 735 (13.35) | x^2^=51.64, df=1, *p*<0.001 |
|  |  |  |  |  |  |
| *Behavioural outcomes* | |  |  |  |  |
|  | Currently a smoker |  | 1072 (17.80) | 974 (14.47) | x^2^=26.02, df=1, *p*<0.001 |

**Supplementary Table 2. Distribution of the cognitive domains across all waves of data collection for the 6817 ELSA participants included in this study**

| **Cognitive domain** | | **Wave 1** | | **Wave 2** | | **Wave 3** | | **Wave 4** | | **Wave 5** | | **Wave 6** | | **Wave 7** | | **Wave 8** |
| --- | --- | --- | --- | --- | --- | --- | --- | --- | --- | --- | --- | --- | --- | --- | --- | --- |
|  |  | Mean (SD) | | Mean (SD) | | Mean (SD) | | Mean (SD) | | Mean (SD) | | Mean (SD) | | Mean (SD) | | Mean (SD) |
|  |  | |  | |  | |  | |  | |  | |  | |  |  |
| Verbal memory | | 10.02 (3.32)  N=5523 | | 10.27 (3.42)  N=5484 | | 10.45 (3.59)  N=5118 | | 10.44 (3.54)  N=5512 | | 10.47 (3.60)  N=5064 | | 10.62 (3.66)  N=4689 | | 10.27 (3.80)  N=4125 | | 9.96 (4.20)  N=3854 |
|  | |  | |  | |  | |  | |  | |  | |  | |  |
| Semantic fluency | | 20.37(6.09)  N=5524 | | 20.44 (6.32)  N=5490 | | 20.45 (6.55)  N=5117 | | 20.91 (6.72)  N=5512 | | 20.93 (6.76)  N=5057 | | - | | 20.99 (7.22)  N=4191 | | 21.20 (7.25)  N=3729 |

SD, standard deviation; ELSA, English Longitudinal Study of Ageing

**Supplementary Table 3. Associations between polygenic score for schizophrenia and longitudinal measure of cognitive function in older adults over the 10-year follow-up, stratified by gender**

|  |  | **Verbal Memory** | | | |  | **Semantic Fluency** | | | |
| --- | --- | --- | --- | --- | --- | --- | --- | --- | --- | --- |
|  |  | **Women** | | **Men** | |  | **Women** | | **Men** | |
|  |  | *β* (95% CI) | *P*-value | *β* (95% CI) | *P*-value |  | *β* (95% CI) | *P*-value | β (95% CI) | *P*-value |
| *Baseline* |  |  |  |  |  |  |  |  |  |  |
| SZ-PGS |  | -0.03 (-0.13-0.08) | 0.630 | -0.12 (-0.23- -0.01) | **0.040** |  | -0.17 (-0.37-0.03) | 0.103 | -0.36 (-0.59- -0.12) | **0.003** |
| Age |  | 0.23 (0.09-0.38) | 0.002 | 0.05 (-0.11-0.22) | 0.503 |  | 0.18 (-0.10-0.46) | 0.207 | .22 (-0.12-0.55) | 0.204 |
| Current smoker |  | -0.10 (-.41-.218 ) | 0.544 | -0.13 (-0.46-0.20 ) | 0.454 |  | -0.47 (-1.08-0.14) | 0.128 | -0.74 (-1.43-0.05) | 0.036 |
| Low level of wealth |  | -0.56 (-0.80-0.33) | <0.001 | -0.56 (-0.82- -0.31 ) | <0.001 |  | -.85 (-1.31- -0.40) | <0.001 | -0.23 (-0.76-0.31) | 0.404 |
| Educational attainment |  | 0.21 (0.17-0.22) | <0.001 | 0.18 (0.15-0.21) | <0.001 |  | 0.50 (0.44- -0.56) | <0.001 | 0.37 (0.30-0.43) | <0.001 |
| Depression diagnosis |  | -0.36 (-0.68- -0.05) | 0.022 | -0.79 (-1.18- -0.40) | <0.001 |  | -0.71 (-1.28- -0.14) | 0.015 | -0.79 (-1.61-.02) | 0.057 |
| *APOE-ε4* present |  | -0.29 (-0.53- -0.04) | 0.020 | -0.26 (-0.51- -0.01) | 0.044 |  | -0.25 (-0.72-0.22) | 0.297 | -0.38 (-0.91-0.15) | 0.157 |
| Limiting health conditions |  | -0.14 (-0.38-0.09) | 0.220 | -0.23 (-0.48-0.02) | 0.077 |  | -0.11 (-0.56-0.34) | 0.638 | -0.31 (-0.84-0.21) | 0.243 |
|  |  |  |  |  |  |  |  |  |  |  |
| *Rate of change* |  |  |  |  |  |  |  |  |  |  |
| SZ-PGS |  | -0.01 (-0.04-0.02) | 0.433 | 0.02 (-0.01-0.04) | 0.148 |  | 0.01 (-0.03-0.04) | 0.802 | 0.004 (-0.04-0.05) | 0.847 |
| Age |  | -0.02 (-0.02- -0.02) | <0.001 | -0.02 (-0.02- -0.01) | <0.001 |  | -0.02 (-0.02- -0.01) | <0.001 | -0.02 (-0.03- -0.02) | <0.001 |
| Current smoker |  | -0.12 (-0.21- -0.02) | 0.015 | -0.06 (-0.13-0.01) | 0.118 |  | -0.05 (-0.17-0.06) | 0.381 | -0.05 (-0.20-0.00) | 0.044 |
| Low level of wealth |  | -0.01 (-.05-.04) | 0.834 | -0.02 (-0.07-0.04) | 0.521 |  | -0.06 (-0.15-0.02) | 0.157 | -.11 (-0.21- -0.003) | 0.044 |
| Educational attainment |  | 0.003 (-0.003-0.01) | 0.344 | 0.001 (-0.01-0.01) | 0.664 |  | -0.002 (-0.01-0.01) | 0.700 | -0.01 (-0.02-0.003) | 0.051 |
| Depression diagnosis |  | -0.04 (-0.10-0.03) | 0.258 | 0.04 (-0.08-0.16) | 0.537 |  | -0.06 (-0.17-0.06) | 0.300 | -0.05 (-0.21-0.11) | 0.531 |
| *APOE-ε4* present |  | -0.09 (-0.14- -0.04) | 0.001 | -0.06 (-0.12-0.01) | 0.016 |  | -0.08 (-0.17-0.01) | 0.070 | -0.10 (-0.20- -0.01) | 0.039 |
| Limiting health conditions |  | -0.05 (-0.10-0.001) | 0.053 | -0.002 (-0.06-0.05) | 0.517 |  | -0.17 (-0.26- -0.09) | <0.001 | -0.04 (-0.14-0.07) | 0.483 |
|  |  |  |  |  |  |  |  |  |  |  |
| *Variance ^a^* |  |  |  |  |  |  |  |  |  |  |
| Within-person |  | 0.08 (0.06-0.09) | | 0.06 (0.046-0.08) | |  | 0.14 (0.10-0.21) | | 0.18 (0.13-0.25) | |
| In initial status |  | 4.07 (3.65-4.54) | | 3.73 (3.33-4.12) | |  | 14.36 (12.78-16.12) | | 17.39 (15.57-19.41) | |
| In rate of change |  | 0.001(-0.068-0.07) | | 0.06(-0.002-0.13) | |  | 0.18 (-0.05-0.41) | | -0.01 (-0.28-0.26) | |

CI, confidence intervals; *APOE-ε4,* two *ε4* alleles of the Apolipoprotein E gene; polygenic score for schizophrenia (SZ-PGS).

The models were further adjusted for age^2^ to capture non-linear aging effects of which cognition is susceptible to and 4 principal components to account for any ancestry differences in genetic structures that could bias the results.

^a^ The within-person variance is the overall residual variance in cognition that is not explained by the model. The initial status variance component is the variance of individuals’ intercepts about the intercept of the average person. The rate of change variance component is the variance of individual slopes about the slope of the average person.

**Supplementary Table 4. Associations between polygenic score for general cognition and longitudinal measure of verbal memory in older adults over the 10-year follow-up, stratified by age groups**

|  |  | **50-59 years** |  | **60-69 years** | |  | **≥70 years** | |  |
| --- | --- | --- | --- | --- | --- | --- | --- | --- | --- |
|  |  | *β* (95% CI) | *P*-value | *β* (95% CI) | | *P*-value | *β* (95% CI) | | *P*-value |
| *Baseline* | |  |  |  | |  |  | |  |
|  | SZ-PGS | -0.04 (-0.17-0.08) | 0.504 | -0.03 (-0.16-0.09) | | 0.590 | -0.14 (-0.28-0.004) | | 0.056 |
|  | Gender | -1.17 (-1.43- -0.91) | <0.001 | -1.22 (-1.47- -0.97) | | <0.001 | -0.83 (-1.12- -0.53) | | 0.000 |
|  | Current smoker | 0.17 (-0.17-0.51) | 0.334 | -0.30 (-0.67-0.06) | | 0.105 | -0.29 (-0.81- 0.22) | | 0.263 |
|  | Low level of wealth | -0.58 (-0.87- -0.28) | <0.001 | -0.67 (-0.95- -0.37) | | <0.001 | -0.42 (-0.74- -0.11) | | 0.008 |
|  | Educational attainment | 0.20 (0.16-0.23) | <0.001 | 0.19 (0.16-0.22) | | <0.001 | 0.20 (0.15-0.24) | | 0.000 |
|  | Depression | -0.48 (-0.87- -0.09) | 0.017 | -.052 (-0.94- -0.09) | | 0.017 | -0.48 (-0.89- -0.06) | | 0.023 |
|  | *APOE-ε4* present | -0.09 (-0.38-0.19) | 0.518 | 0.01 (-0.28-0.29) | | 0.971 | -0.82 (-1.16- -0.48) | | 0.000 |
|  | Limiting health conditions | -0.30 (-0.61-0.004) | 0.053 | -0.33 (-0.61- -0.04) | | 0.024 | 0.13 (-0.17-0.44) | | 0.387 |
|  |  |  |  |  | |  |  | |  |
| *Rate of change* | |  |  |  | |  |  | |  |
|  | SZ-PGS | 0.0001 (-0.02-0.02) | 0.992 | 0.01 (-0.02-0.03) | | 0.622 | 0.01 (-0.03-0.04) | | 0.759 |
|  | Gender | -0.02 (-0.07-0.03) | 0.362 | -0.05 (-0.10-0.001) | | 0.054 | 0.06 (-0.02-0.13) | | 0.137 |
|  | Current smoker | -0.04 (-0.11-0.03) | 0.279 | -0.11 (-0.18- -0.03) | | 0.008 | -0.03 (-0.17-0.12) | | 0.727 |
|  | Low level of wealth | -0.02 (-0.08-0.04) | 0.558 | 0.01 (-0.04-0.07) | | 0.632 | -0.03 (-0.11-0.05) | | 0.506 |
|  | Educational attainment | 0.01 (-0.001-0.01) | 0.115 | 0.01 (-0.001-0.01) | | 0.092 | -0.01 (-0.02-0.004) | | 0.184 |
|  | Depression diagnosis | 0.06 (-0.014-0.14) | 0.110 | -0.02 (-0.10 0.07) | | 0.706 | -0.13 (-0.24- -0.02) | | 0.016 |
|  | *APOE-ε4* present | -0.07 (-0.13- -0.02) | 0.011 | -0.08 (-0.14- -0.03) | | 0.004 | -0.09 (-0.18- 0.00) | | 0.050 |
|  | Limiting health conditions | -0.03 (-0.090-.03) | 0.397 | -0.03 (-0.09-0.02) | | 0.258 | -0.03 (-0.11-0.05) | | 0.468 |
|  |  |  |  |  | |  |  | |  |
| *Variance ^a^* | |  |  |  | |  |  | |  |
|  | Within-person | 0.06 (0.05-0.08) | | | 0.06 (0.05-0.08) | | | 0.10 (0.07-0.13) | |
|  | In initial status | 3.47 (3.02-3.40) | | | 3.54 (3.10-4.05) | | | 4.56 (3.95-5.26) | |
|  | In rate of change | -0.01 (-.095-0.059) | | | 0.11 (0.04-0.19) | | | -0.03 (-0.15-0.09) | |

CI, confidence intervals; *APOE-ε4,* two *ε4* alleles of the Apolipoprotein E gene; polygenic score for schizophrenia (SZ-PGS)

The models were further adjusted for age^2^ to capture non-linear aging effects of which cognition is susceptible to and 4 principal components to account for any ancestry differences in genetic structures that could bias the results.

^a^The within-person variance is the overall residual variance in memory that is not explained by the model. The initial status variance component is the variance of individuals’ intercepts about the intercept of the average person. Likewise, the rate of change variance component is the variance of individual slopes about the slope of the average person.

**Supplementary Table 5. Associations between polygenic score for general cognition and longitudinal measure of semantic fluency in older adults over the 10-year follow-up, stratified by age groups**

|  |  | **50-59 years** |  | **60-69 years** | |  | **≥70 years** | |  |
| --- | --- | --- | --- | --- | --- | --- | --- | --- | --- |
|  |  | *β* (95% CI) | *P*-value | *β* (95% CI) | | *P*-value | *β* (95% CI) | | *P*-value |
| *Baseline* | |  |  |  | |  |  | |  |
|  | SZ-PGS | -0.25 (-0.53-0.03) | 0.080 | -0.32 (-0.58- -0.05) | | **0.019** | -0.17 (-0.41-0.08) | | 0.193 |
|  | Gender | -0.39 (-0.94- -0.17) | 0.177 | 0.03 (-0.50-0.57) | | 0.900 | 0.31 (-0.20-0.82) | | 0.234 |
|  | Current smoker | -0.32 (-1.07-0.43) | 0.407 | -0.68 (-1.47-0.10) | | 0.087 | -0.95 (-1.84- -0.06) | | 0.036 |
|  | Low level of wealth | -0.11 (-0.75-0.54) | 0.745 | -0.61 (-1.24-0.01) | | 0.054 | -0.97 (-1.51- -0.42) | | 0.001 |
|  | Educational attainment | 0.50 (0.42-0.57) | 0.000 | 0.43 (0.35-0.50) | | <0.001 | 0.37 (0.30-0.45) | | 0.000 |
|  | Depression | -0.97 (-1.82- -0.11) | 0.027 | -0.28 (-1.19-0.62) | | 0.540 | -0.95 (-1.66- -0.24) | | 0.009 |
|  | *APOE-ε4* present | -0.28 (-0.91-0.35) | 0.387 | -0.25 (-0.86-0.36) | | 0.415 | -0.42 (-1.01-0.17) | | 0.168 |
|  | Limiting health conditions | -0.62 (-1.29-0.05) | 0.070 | -0.01 (-0.62-0.60) | | 0.966 | -0.01 (-0.534- 0.51) | | 0.965 |
|  |  |  |  |  | |  |  | |  |
| *Rate of change* | |  |  |  | |  |  | |  |
|  | SZ-PGS | -0.01 (-0.05-0.04) | 0.869 | 0.01 (-0.04-0.06) | | 0.696 | 0.02 (-0.05-0.09) | | 0.535 |
|  | Gender | -0.01 (-0.10-0.08) | 0.879 | -0.09 (-0.18-0.01) | | 0.070 | -0.04 (-0.17-0.10) | | 0.588 |
|  | Current smoker | -0.07 (-0.19-0.05) | 0.244 | -0.05 (-0.20-0.09) | | 0.482 | 0.08 (-0.19-0.35) | | 0.573 |
|  | Low level of wealth | -0.06 (-0.16-0.05) | 0.300 | -0.14 (-0.25- -0.02) | | 0.017 | -0.02 (-0.17-0.13) | | 0.805 |
|  | Educational attainment | -0.02 (-0.05-0.01) | 0.177 | 0.002 (-0.01-0.01) | | 0.812 | -0.02 (-0.03-0.002) | | 0.079 |
|  | Depression diagnosis | -0.04 (-0.18-0.10) | 0.563 | -0.07 (-0.23-0.09) | | 0.405 | -0.09 (-0.28-0.11) | | 0.377 |
|  | *APOE-ε4* present | -0.06 (-0.14-0.01) | 0.134 | -0.15 (-0.28- -0.03) | | .014 | -0.16 (-0.32-0.004) | | 0.056 |
|  | Limiting health conditions | -0.04 (-0.14-0.07) | 0.495 | -0.20 (-0.30- -0.09) | | <0.001 | -0.08 (-0.22-0.07) | | 0.299 |
|  |  |  |  |  | |  |  | |  |
| *Variance ^a^* | |  |  |  | |  |  | |  |
|  | Within-person | 0.09 (0.05-0.17) | | | 0.18 (0.13-.25) | | | 0.28 (0.19-0.40) | |
|  | In initial status | 17.29 (15.13-19.76) | | | 17.54 (15.46-19.90) | | | 12.64 (10.73-14.90) | |
|  | In rate of change | 0.10 (-0.19-0.39) | | | -0.06 (-0.35-0.23) | | | 0.13 (-0.24-0.51) | |

CI, confidence intervals; *APOE-ε4,* two *ε4* alleles of the Apolipoprotein E gene; polygenic score for schizophrenia (SZ-PGS)

The models were further adjusted for age^2^ to capture non-linear aging effects of which cognition is susceptible to and 4 principal components to account for any ancestry differences in genetic structures that could bias the results.

^a^The within-person variance is the overall residual variance in memory that is not explained by the model. The initial status variance component is the variance of individuals’ intercepts about the intercept of the average person. Likewise, the rate of change variance component is the variance of individual slopes about the slope of the average person.

**Supplementary Figure 1. Depicts distribution of 10 principal components once 65 individuals with ancestral admixture were removed from the sample.**


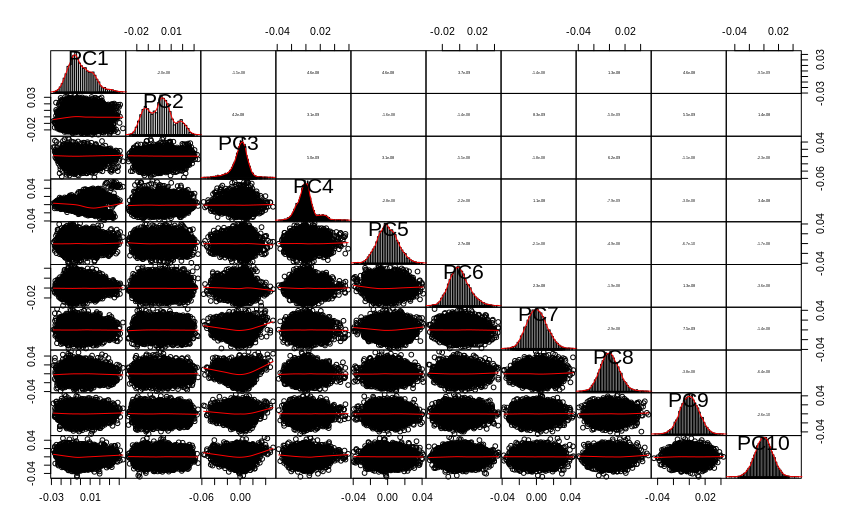

Supplement: Supplementary file 1 — Supplementary material [file 41398_2020_1114_MOESM1_ESM.docx]
